# Molecular Signatures of Inflammatory Profile and B-Cell Function in Patients with Severe Fever with Thrombocytopenia Syndrome

**DOI:** 10.1128/mBio.02583-20

**Published:** 2021-02-16

**Authors:** Angela Park, Su-Jin Park, Kyle L. Jung, Se Mi Kim, Eun-Ha Kim, Young-Il Kim, Suan-Sin Foo, Sunghyun Kim, Seong-Gyu Kim, Kwang-Min Yu, Younho Choi, Ji Yeun Kim, Yun Hee Baek, Min-Suk Song, Seung Ryul Kim, Seok-Yong Kim, Hye Won Jeong, Sung-Han Kim, Jae U. Jung, Young Ki Choi

**Affiliations:** a Department of Molecular Microbiology and Immunology, Keck School of Medicine, University of Southern California, Los Angeles, California, USA; b College of Medicine and Medical Research Institute, Chungbuk National University, Cheongju, Republic of Korea; c Zoonotic Infectious Diseases Research Center, Chungbuk National University, Cheongju, Republic of Korea; d Department of Cancer Biology and Global Center for Pathogens Research and Human Health, Lerner Research Institute, Cleveland Clinic, Cleveland, Ohio, USA; e Department of Infectious Diseases, Asan Medical Center, University of Ulsan College of Medicine, Seoul, Republic of Korea; f Division of Life Science, Research Institute of Life Science, Gyeongsang National University, Jinju, Republic of Korea; Johns Hopkins Bloomberg School of Public Health

**Keywords:** emerging virus, bandavirus, SFTSV, SFTS, immunoprofiling, proximity extension assay, single-cell RNA-seq, plasma B cell

## Abstract

Dabie bandavirus (severe fever with thrombocytopenia syndrome virus [SFTSV]) induces an immunopathogenic disease with a high fatality rate; however, the mechanisms underlying its clinical manifestations are largely unknown. In this study, we applied targeted proteomics and single-cell transcriptomics to examine the differential immune landscape in SFTS patient blood. Serum immunoprofiling identified low-risk and high-risk clusters of SFTS patients based on inflammatory cytokine levels, which corresponded to disease severity. Single-cell transcriptomic analysis of SFTS patient peripheral blood mononuclear cells (PBMCs) at different infection stages showed pronounced expansion of B cells with alterations in B-cell subsets in fatal cases. Furthermore, plasma cells in which the interferon (IFN) pathway is downregulated were identified as the primary reservoir of SFTSV replication. This study identified not only the molecular signatures of serum inflammatory cytokines and B-cell lineage populations in SFTSV-induced fatalities but also plasma cells as the viral reservoir. Thus, this suggests that altered B-cell function is linked to lethality in SFTSV infections.

## INTRODUCTION

Since the disease was first reported in 2009, the number of cases of severe fever with thrombocytopenia syndrome (SFTS), caused by a novel bandavirus of the *Bunyaviridae* order (1), has rapidly increased (2–4). SFTS is characterized by high fever, thrombocytopenia, leukocytopenia, gastrointestinal symptoms, hemorrhage, and multiple-organ failure with high fatality. Although the average fatality rate varies among regions, the mean mortality rates of SFTS have remained relatively high in Japan (27%), South Korea (23.3%), and China (5.3 to 16.2%) (3, 5, 6). However, the mechanisms underlying the differential mortality rates and clinical manifestations are largely unknown. Several studies have compared host factors such as age, serum cytokine profile, and peripheral blood mononuclear cell (PBMC) composition with clinical manifestations (7–10). In severe cases of infection, serum cytokines are significantly upregulated, suggesting that the interaction between the virus and the host immune system plays an important role in determining the outcome of SFTS virus (SFTSV) infection (11–13). Recent studies also showed that SFTSV effectively infects monocytes, which interferes with innate immune signaling pathways (14, 15). Further, Song et al. reported that SFTS fatality is associated with the absence of virus-specific B-cell immunity and a low IgG antibody titer, suggesting a critical role of B-cell function in disease outcome (7). Despite these observations, the exact mechanisms underlying the differential clinical manifestations remain elusive. To investigate the association between viral pathogenesis and host immunity, a better understanding of the immune landscape and gene expression profile in SFTS patients at various disease states is required.

A previous study showed that degrees of SFTSV seroprevalence in healthy people are not significantly different among age groups, but all clinically diagnosed SFTS patients were older than ∼50 years (16, 17). In fact, patients ≥70 years of age showed the highest mortality rate, followed by patients 60 to 69 years old and 50 to 59 years old (17). The fact that aged people have weakened immune systems suggests that immunosenescence is a critical risk factor and determinant for SFTSV morbidity and mortality (18).

The evaluation of blood‐based biomarkers for the diagnosis of SFTS is desirable due to the inaccessibility of disease‐targeted tissue sampling, especially in cases of severe infection (13, 19). Therefore, accurate, sensitive, and reproducible methods are needed to identify biomarkers and provide molecular insight into the severity of SFTS during diagnosis and progression.

Since the immune status of infected individuals may play an important role in determining disease severity and clinical outcomes, we applied precision proteomics and single-cell transcriptomics to analyze the differential immune modulation in SFTS patients. A novel nucleic acid-based proximity extension assay (PEA) technology developed by Olink Bioscience (Uppsala, Sweden) (20) affords an advantage over conventional multiplex immunoassays, as it can provide accurate quantification at levels below picogram per milliliter, even in a small quantity of patient serum. In addition, single-cell transcriptome sequencing (scRNA-seq) offers an opportunity to investigate the individual transcriptomes of tens of thousands of single cells simultaneously, without a limit on the number of genes that can be used for analysis (21). In this study, we applied PEA-derived immunoprofiling and scRNA-seq analyses to characterize SFTS patient blood. The results of serum immunoprofiling of SFTS patients were found to reflect the severity of clinical symptoms. scRNA-seq analysis showed a unique expansion of the B-cell population in SFTSV-induced fatal cases and also indicated that plasma B cells are a primary reservoir of SFSTV replication. This suggests that the PEA-derived inflammatory signature and scRNA-seq-derived immune cell landscape contain critical biomarkers of SFTS disease progression and outcome.

## RESULTS

### Serum immunoprofiling of SFTSV-positive patients identifies inflammatory signatures associated with fatality.

**FIG 1:**
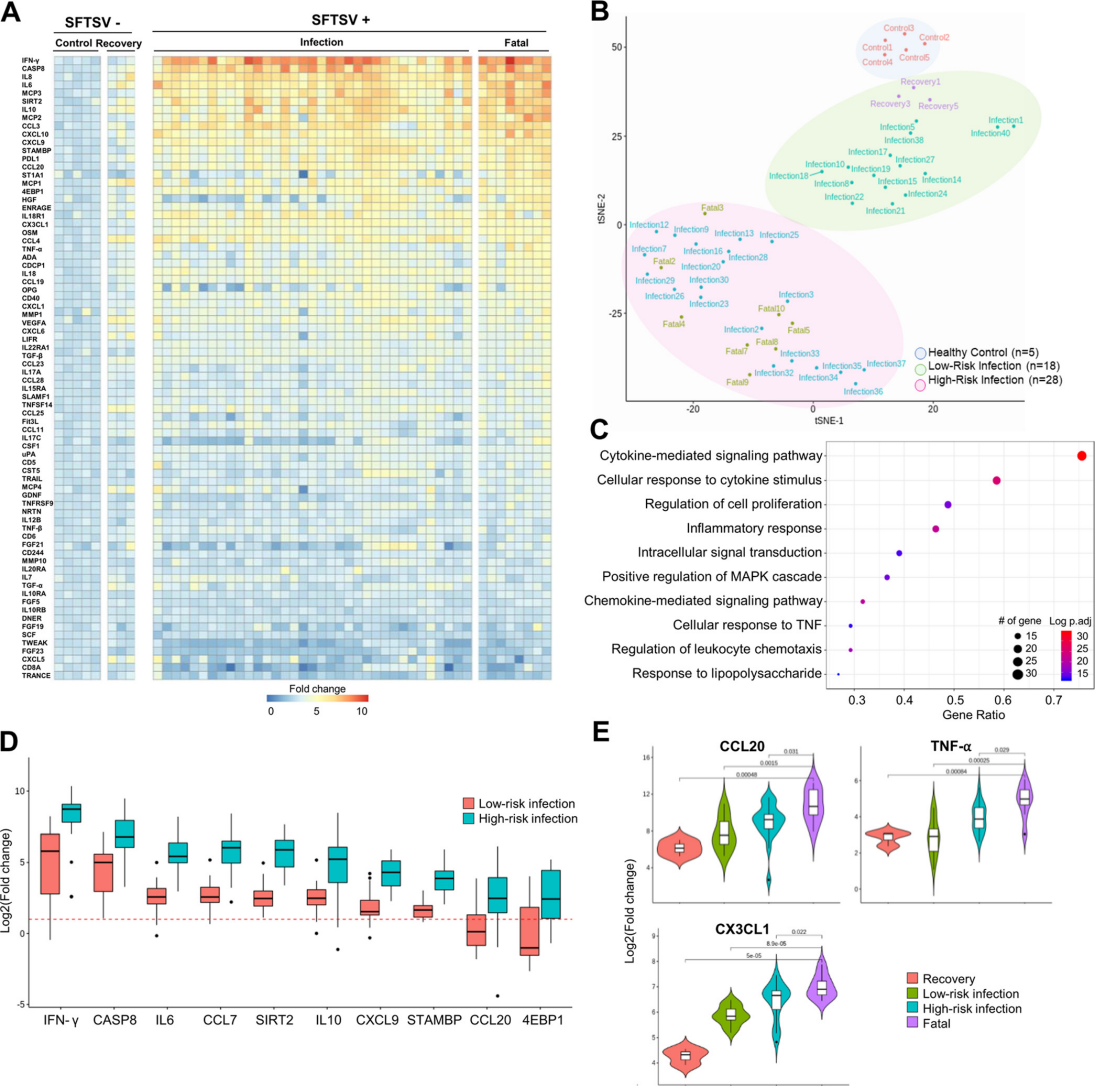
Differential serum immunoprofiles of SFTSV patients. Shown is multiplex immune profiling of 76 inflammatory molecules using serum specimens derived from 5 healthy age-matched controls and 46 SFTSV^+^ patients (35 infection, 3 recovery, and 8 fatality). (A) Heat map of 76 inflammatory cytokines and chemokines in the SFTSV^−^ cohort (control and recovery) and SFTSV^+^ cohort (infection and fatality). Each box color represents fold change relative to the healthy control group. Each column represents a patient and each row represents a soluble factor measured by the immunoassay ordered in decreasing significance. (B) *t*-distributed stochastic neighbor embedding (*t*-SNE) plot showing unbiased clustering of the cohort based on cytokine and chemokine levels. Based on proximity, samples were subcategorized into healthy control, low-risk infection, and high-risk infection. The healthy control cluster includes 5 healthy age-matched controls, the low-risk infection cluster includes 3 recovery patients and 15 infection patients, and the high-risk infection cluster includes fatal cases and 20 infection patients. (C) Top 10 gene ontology (GO) biological pathway enrichment analysis for cytokines and chemokines induced in SFTSV^+^ patients. The *x* axis represents the ratio of enrichment (the number of observed genes divided by the number of expected genes from each GO in the gene list), and the *y* axis represents the enriched pathway in the GO database. The size of circles indicates the number of genes, and the color indicates adjusted log *P* value. (D) Fold changes of top 10 significantly altered cytokines and chemokines (*P < *0.05) in low-risk and high-risk groups compared to the control group. The red dashed line represents the control value (basal level of 1). (E) Top three cytokines and chemokines associated specifically with fatal cases. *P* values are written above each bracket.

To characterize SFTSV-mediated immune responses, we assayed 76 soluble inflammatory factors in patient sera using a proximity extension assay (PEA). Groups analyzed were age-matched healthy men and women (control group), SFTS patients directly after hospitalization (infection and fatal groups), and patients after at least 2 weeks of discharge (recovery group) from the hospital. The infection and fatal groups showed considerable increases in several inflammatory factors (gamma interferon [IFN-γ], interleukin 6 [IL-6], IL-8, SIRT2, CCL3, and CXCL10) compared to the control group (Fig. 1A). On the other hand, the recovery group showed minor changes relative to the control group, but several inflammatory factors, including IL-8, SIRT2, MCP1, CCL4, MMP-1, TNFS14, and MCP4, were still at higher levels in the patient sera of the recovery group than in those of the control group, suggesting sustained inflammation despite viral clearance. A *t*-distributed stochastic neighbor embedding (*t*-SNE) analysis of patient serum immunoprofiling strikingly showed three distinct, unbiased clusters of differential inflammatory factor levels (Fig. 1B). While the control group clustered as its own group, the infection groups were divided into two subgroups, the infection/recovery group and infection/fatal group, which we refer to as low risk and high risk, respectively. Gene ontology (GO) revealed that the infected groups (infection and fatal groups) showed activation of inflammatory cytokine/chemokine signaling pathways as well as cell proliferation pathways compared to the control group (Fig. 1C). The top 10 inflammatory factors that were significantly altered in the high-risk group but not in the low-risk group were IFN-γ, CASP8, IL-6, MCP3 (CCL7), SIRT2, IL-10, CXCL9, STAMBP, CCL20, and 4EBP1 (Fig. 1D). Thus, this unique immune signature is specific for severe infection cases. Lastly, we also identified three inflammatory factors, CCL20, tumor necrosis factor alpha (TNF-α), and CX3CL1, as potential serum markers uniquely associated with fatality, as they were considerably increased in sera from all 8 of the fatal cases compared to those of the rest of the groups (Fig. 1E). Moreover, several of these identified markers were confirmed via multiplex enzyme-linked immunosorbent assay (ELISA) (see Fig. S1A to H). In summary, serum immunoprofiling of SFTS patients revealed that infected patients can be divided into low-risk and high-risk groups (where all fatal cases belong to the high-risk group) based on inflammatory protein expression. Of these, 10 serum proteins are associated with severe infection and three are associated with fatal cases.

10.1128/mBio.02583-20.1 FIG S1 Cytokine and chemokine levels in sera from control and SFTSV^+^ patients. A multiplex biometric immunoassay was conducted with the Bio-Plex Pro human cytokine 17-plex immunoassay kit (Bio-Rad; M50-00031YV). Concentrations of each cytokine and chemokine in sera of SFTSV infected, recovery, fatal, and control groups are shown. CCL2 (A), CCL4 (B), IL-8 (C), TNF-α (D), IL-6 (E), granulocyte colony-stimulating factor (G-CSF) (F), IFN-γ (G), and IL-10 (H) were measured by multiplex bead-based Bioplex assay (Bio-Rad, Hercules, CA). The concentration of each cytokine and chemokine is shown as the mean ± SEM. The detailed numbers for each group were as follows: age-matched healthy control group, *n *= 6; SFTSV infection group, *n *= 35; recovery group, *n *= 3; and fatal group, *n *= 8. The immunoassay tested for the TNF-α biomarker, associated with fatality, and for IL-6, G-CSF, IFN-γ, and IL-10, which are associated with high-risk infection. Asterisks indicate the statistical significance determined by one-way ANOVA with Tukey’s multiple-comparison test (*, *P < *0.05; **, *P < *0.01; ***, *P < *0.0001). Download FIG S1, TIF file, 0.4 MB. Copyright © 2021 Park et al. 2021 Park et al. https://creativecommons.org/licenses/by/4.0/ This content is distributed under the terms of the Creative Commons Attribution 4.0 International license.

To compare the protein expression-based clustering of the SFTS patient risk assessment with clinical manifestations, we developed an arbitrary scoring method rather than precise clinical data to differentiate the SFTS patients into low-risk and high-risk groups based on available clinical records (*n *= 44), as described in Table 1. The low-risk group showed disease scores of ≤5 (mean score ± standard deviation [SD] = 4.0 ± 1) with clinical symptoms common in SFTSV patients: high fever, thrombocytopenia, and leukocytopenia. Patients in the high-risk infection group showed disease scores of more than 6 (mean score ± SD = 7.1 ± 1.0) with severe clinical symptoms and high virus isolation rates. Interestingly, with exception of three patients, the PEA-derived serum immunoprofiling of low-risk and high-risk groups reflected the severity of clinical symptoms in these groups. Thus, this suggests that the PEA-derived inflammatory signatures may be potential biomarkers of SFTS disease progression and outcome.

**TABLE 1:** Baseline demographics and clinical scoring of cohorts included in the precision proteomics study^*a*^

Parameter	% (mean score ± SD)				
	Control (*n* = 5)	Infection			Recovery (*n* = 3)
		Low risk (*n* = 12)	High risk		
			Survival (*n* = 16)	Deceased (*n* = 8)	
Age (≥60 yrs)	100 (2 ± 0)	50 (1.67 ± 0.78)	87.5 (2.5 ± 0.73)	100 (2.25 ± 0.43)	66.6 (1.3 ± 0.6)
High fever (≥38°C)	0 (0 ± 0)	75 (0.75 ± 0.45)	75 (0.8 ± 0.41)	75 (0.86 ± 0.38)	0 (0 ± 0)
Thrombocytopenia	0 (0 ± 0)	50 (0.5 ± 0.52)	100 (1.0 ± 0)	100 (1 ± 0)	0 (0 ± 0)
Leukocytopenia	0 (0 ± 0)	50 (0.5 ± 0.52)	68.8 (0.73 ± 0.43)	75 (0.86 ± 0.38)	0 (0 ± 0)
Hemophagocytosis	0 (0 ± 0)	0 (0 ± 0)	0 (0 ± 0)	25 (0.5 ± 0.92)	0 (0 ± 0)
Septic shock	0 (0 ± 0)	0 (0 ± 0)	6.3 (0.06 ± 0.25)	0 (0 ± 0)	0 (0 ± 0)
Meningoencephalitis	0 (0 ± 0)	8.3 (0.08 ± 0.23)	6.3 (0.06 ± 0.25)	0 (0 ± 0)	0 (0 ± 0)
Gastrointestinal symptoms	0 (0 ± 0)	16.7 (0.17 ± 0.39)	37.5 (0.3 ± 0.5)	25 (0.25 ± 0.46)	0 (0 ± 0)
Seizure	0 (0 ± 0)	0 (0 ± 0)	6.3 (0.06 ± 0.25)	0 (0 ± 0)	0 (0 ± 0)
Multiorgan dysfunction	0 (0 ± 0)	0 (0 ± 0)	0 (0 ± 0)	50 (0.5 ± 0.53)	0 (0 ± 0)
Rash	0 (0 ± 0)	8.3 (0.08 ± 0.29)	0 (0 ± 0)	0 (0 ± 0)	0 (0 ± 0)
ICU admission	0 (0 ± 0)	16.7 (0.17 ± 0.39)	50 (0.5 ± 0.52)	100 (1.0 ± 0)	0 (0 ± 0)
Virus isolation	0 (0 ± 0)	8.3 (0.08 ± 0.29)	56.3 (0.56 ± 0.51)	100 (1.0 ± 0)	0 (0 ± 0)
Total mean score ± SD	2 ± 0	4.0 ± 1.0	6.6 ± 0.8	8.0 ± 0.8	1.3 ± 0.6

a Score is calculated based on each symptom: +1 , age < 60, high fever (≥38°C), thrombocytopenia, leukocytopenia, hemophagocytosis examination, septic shock, meningoencephalitis, gastrointestinal symptoms, seizure, multiorgan dysfunction, rash, intensive care unit (ICU) admission, virus isolation; +2, 60 ≤ age < 70, hemophagocytosis positive; and +3, age ≥ 70. Low-risk infection group ≤ score of 5; high-risk infection group > score of 5. This group is subdivided into surviving and deceased.

### Single-cell transcriptomics reveals SFTSV-induced global alteration of the host immune landscape.

**FIG 2:**
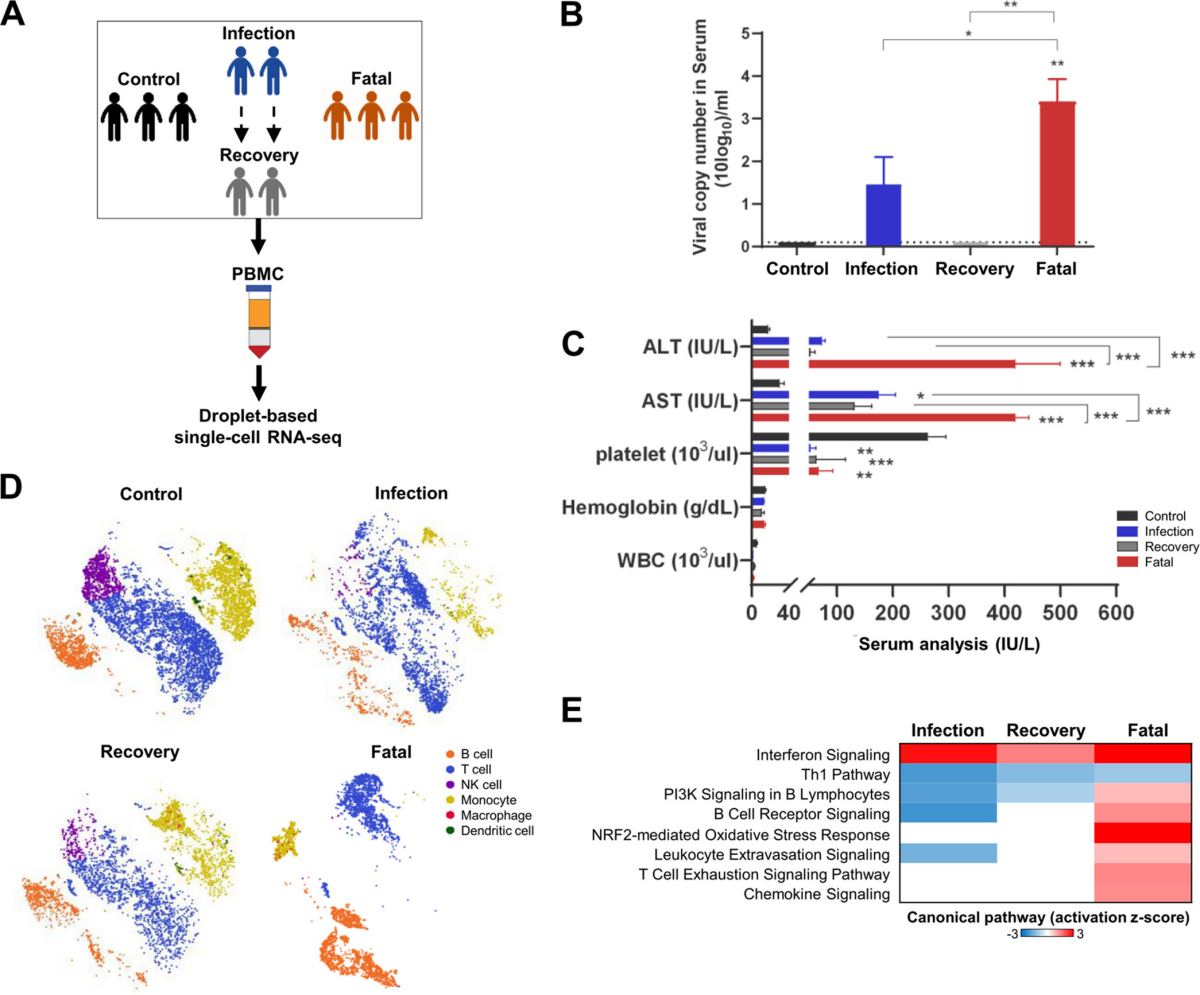
Changes in the host immune landscape upon SFTSV infection. (A) Schematic diagram of single-cell transcriptome study. Blood samples were collected from 8 patients: 3 healthy age-matched control patients (control group), 2 patients with SFTSV infection during the infection and after viral clearance (infection group and recovery group, respectively), and 3 patients who died from SFTSV infection (fatal group). PBMCs were isolated from blood for scRNA-seq analysis. (B) Quantification of SFTSV copy numbers in patient sera using qPCR. The viral copy numbers in sera were higher in the fatal group (3.5 to 4.8 log_10_ copies/ml) than in the infection group (2.8 to 3.5 log_10_ copies/ml). (C) Quantification of liver enzymes (ALT and AST), platelets, hemoglobin, and white blood cells (WBC). Blood samples were subjected to hematological examination using a Celltac hematology analyzer. The average values of ALT, AST, platelets, hemoglobin, and white blood cells were 17.7, 30, 262.7, 14.5, and 5.3 in the control group, 73, 174.5, 52.5, 13.1, and 1.5 in the infection group, 53, 131.5, 63, 10.6, and 3 in the recovery group, and 419.7, 419.7, 67, 13.7, and 1.9 in the fatal group. Asterisks indicate statistical significance between the control and infection groups or between the two groups indicated by the line determined by one-way ANOVA and subsequent Dunnett’s test (*, *P* < 0.05; **, *P < *0.001; ***, *P < *0.0001). (D) *t*-SNE visualization of PBMC populations. Individuals were divided into four groups based on infection status. Clusters are characterized by expression of markers defined in Fig. S2D. (E) Comparison analysis of canonical pathways significantly enriched in each infection group compared to the control group. A negative z score means inactivation of genes in a pathway compared to the control group, and a positive z score means activation of a gene in a pathway compared to the control group.

To investigate how the virus regulates host genes at various stages of SFTS, we performed single-cell transcriptome sequencing (scRNA-seq). We collected peripheral blood mononuclear cell (PBMC) specimens from 8 donors: 3 age-matched healthy men (*n = *2) and women (*n = *1) (control group), 2 SFTS patients before and after viral clearance (infection group and recovery group, respectively), and 3 SFTS patients who succumbed to infection (fatal group) (Fig. 2A). SFTSV infection was confirmed in the collected samples by reverse transcription-PCR (RT-PCR) as previously described (22). Viral copy numbers in sera were significantly higher in the fatal group than in the infection group (Fig. 2B). Hematological analysis revealed that platelets were drastically depleted in the infection, recovery, and fatal groups compared to those in the control group, and the alanine aminotransferase (ALT) and aspartate aminotransferase (AST) levels in sera were markedly high only in the fatal group (Fig. 2C). These results demonstrate clear differences in viral titers and hematological parameters based on disease severity among tested SFTS patients.

10.1128/mBio.02583-20.2 FIG S2 10x quality control (QC) and PBMC subtype determination and quantification. (A) Plots of quality control metrics, which include number of genes, number of unique molecular identifiers (UMIs), and percentage of transcripts mapping to the mitochondrial genome, for each sample. Cells with more than 10% mitochondrial transcript percentage of total UMIs were excluded. (B) *t*-SNE plot of unsupervised clustering of the final 25,802 cells after mitochondrial filtering. (C) *t*-SNE plots of the cells used in the analysis split by patient (3 control, 2 infection, 2 recovery, and 3 fatal). (D) Determination of each lymphocyte subtype by gene expression profiles shown below each subtype. Expression of at least two gene markers was used to identify specific lymphocyte subtypes of the initial 15 cell clusters. Macrophage and dendritic cell types were defined by picking monocytes with the expression of CD163 plus SIGLEC1 and FCER1A plus CST3, respectively. (E) *t*-SNE plot showing 25,802 cells in the analysis colored by defined lymphocyte subtypes. Download FIG S2, PDF file, 0.4 MB. Copyright © 2021 Park et al. 2021 Park et al. https://creativecommons.org/licenses/by/4.0/ This content is distributed under the terms of the Creative Commons Attribution 4.0 International license.

PBMCs from each group were subjected to scRNA-seq using 10x Genomics Chromium, and all data were merged to perform unsupervised clustering based on gene expression. Cells were then visualized in 2-dimensional space using *t*-SNE (Fig. S2A and C) based on their projection and labeled as different PBMC subtypes: B cells, T cells, NK cells, monocytes, macrophages, and dendritic cells (Fig. 2D and Fig. S2D and E). In comparison to the control group, the recovery and infection groups showed small changes in immune cell clustering, while the fatal group exhibited dramatic rearrangements, indicating considerably different gene expression profiles of immune cells between the fatal group and the rest of the groups. Ingenuity pathway analysis (IPA) of total gene expression indicated that the interferon (IFN) signaling pathway was highly activated in all SFTSV-infected groups (infection, recovery, and fatal groups) compared to the control group, as shown by high positive z scores (predicted activation) (Fig. 2E). Interestingly, only the fatal group showed significant activation of adaptive immune response signaling pathways, including B-cell receptor (z score = 1.342), leukocyte extravasation (z score = 0.816), and T-cell exhaustion signaling (z score = 1.414). Taken together, the results show that SFTSV infection led to the activation of the innate IFN pathway at various stages of disease progression, whereas adaptive immune responses were induced primarily in patients who later succumbed to the infection.

### SFTSV-induced fatality is associated with robust B-cell expansion and immune activation.

**FIG 3:**
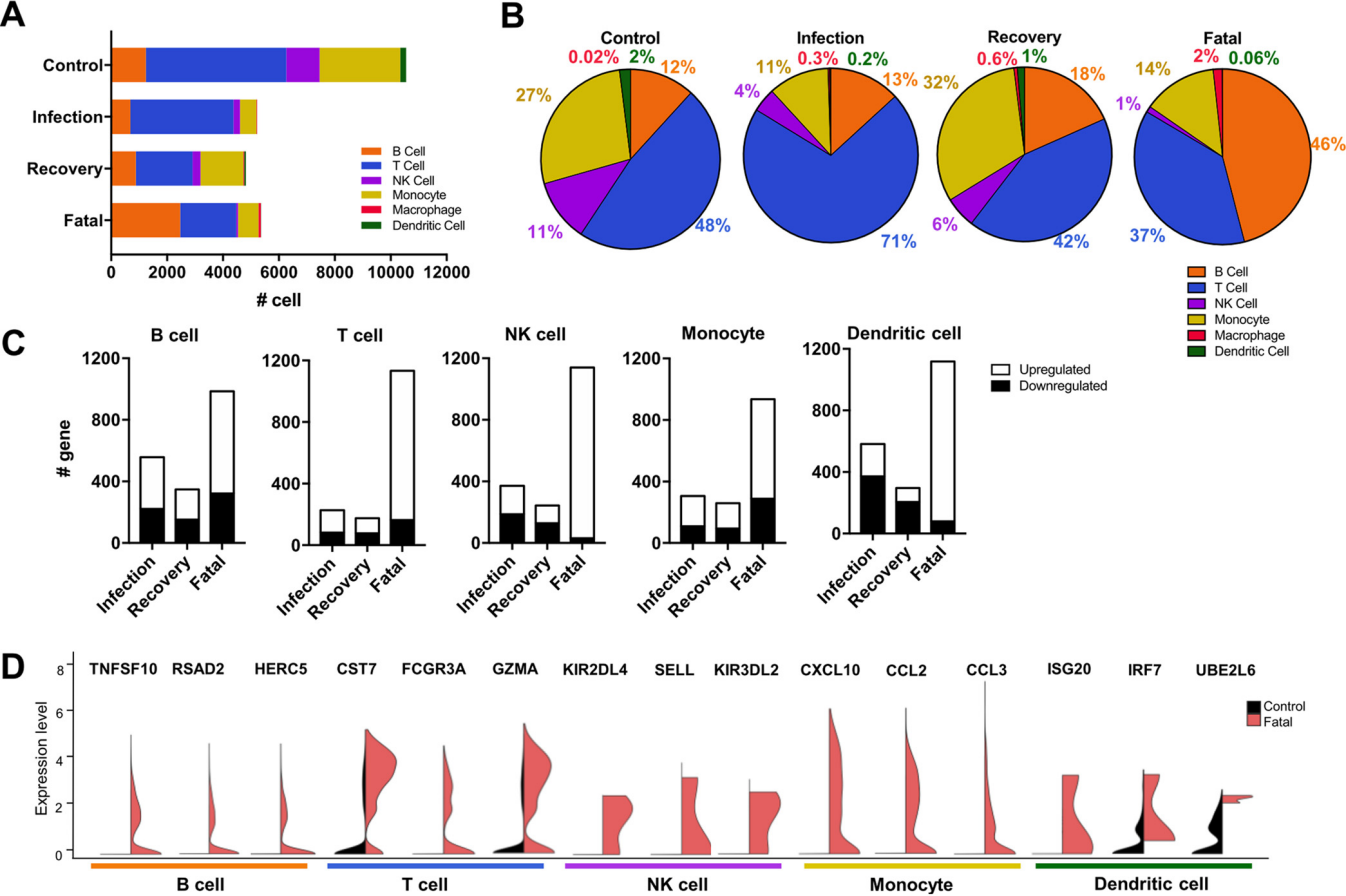
B-cell expansion and overt immune activation in fatal SFTSV infection. (A) Bar chart showing absolute count of each lymphocyte population in PBMCs. Colors in the bars represent total values for the respective cell populations. (B) Pie chart showing percentages of each lymphocyte population in total PBMCs. (C) Total number of significantly upregulated (white) or downregulated (black) genes in PBMCs of each patient group compared to the control group (*P* < 0.05 and fold change > 2). (D) Split violin plots showing highly expressed genes of each lymphocyte population in patients who died from SFTSV infection. Three gene markers are shown for each PBMC subtype.

To gain a full understanding of the lymphocyte composition of SFTS patient PBMCs, we calculated absolute numbers and percentages of each cell type based on the *t*-SNE-based subcategorization (Fig. 3A). Although the recovery group showed lower absolute cell numbers by scRNA-seq, the composition and clustering outlook were very similar to those of the control group, further confirming the similarity of the immune profiles between the control and the recovery groups (Fig. 3B). Disease severity was negatively correlated with NK cell and dendritic cell counts but positively correlated with the circulating-macrophage count (Fig. 3B). The monocyte population in the infection group was considerably reduced compared to that in the control group, while it was recovered in the recovery group. In addition, B- and T-cell populations exhibited more dynamic changes than other lymphocyte populations. While the total PBMCs of the infection and recovery groups showed B-cell percentages similar to that of the control group (12 to 18%), this B-cell proportion was dramatically increased, to 46%, in the fatal group. Also, the T-cell population was expanded to 71% of total PBMCs in the infection group, while it was maintained at 37 to 48% in other groups (Fig. 3B).

To uncover gene expression patterns at different disease stages of SFTS, we compared differentially expressed genes (DEGs) in PBMC subtypes from SFTS patient groups with those of the control group (Fig. 3C). Similar numbers of genes were significantly upregulated or downregulated in every cell type of the infection group (Fig. 3C). Strikingly, the fatal group showed the highest number of DEGs in every cell type, with the majority being upregulated (Fig. 3C). Among these genes, the top three genes from each PBMC subtype, which were altered specifically in the fatal group and not in the infection or recovery group, were TNFSF10 (Trail), RSAD2 (viperin), and HERC5 in B cells, CST7, FCGR3A, and GZMA in T cells, killer cell immunoglobulin-like receptors (KIRs) and SELL in NK cells, CXCL10, CCL2, and CCL3 in monocytes, and type I IFN-inducible ISG20, IRF7, and UBE2L6 in dendritic cells (Fig. 3D). Collectively, compared to the control, infection, and recovery groups, the fatal group exhibited pronounced expansion of the B-cell population and increased expression of immunity-related genes in all blood cell subsets.

### B cells are the primary viral reservoir in cases of fatal SFTSV infection.

**FIG 4:**
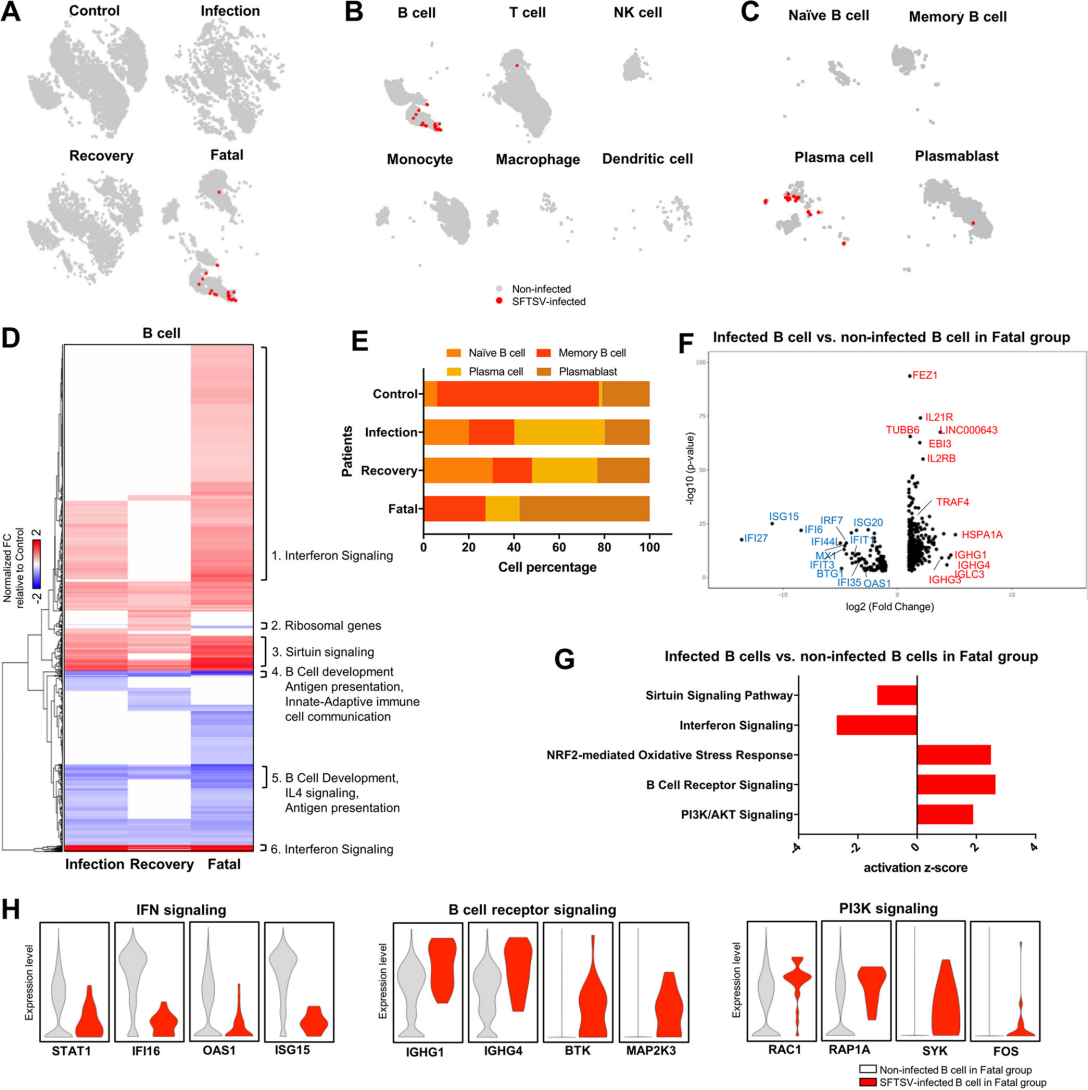
B cells are the primary viral reservoir in fatal cases of SFTSV infection. (A) *t*-SNE plots of all four sample groups. Cells with SFTSV transcripts are colored red, while cells without SFTSV transcripts are colored gray. (B) *t*-SNE plots of different lymphocyte populations with SFTSV^+^ cells (red) and SFTSV^−^ cells (gray) in lethal SFTSV infections. (C) *t*-SNE plots of B-cell subtypes with SFTSV^+^ cells (red) and SFTSV^−^ cells (gray) in lethal SFTSV infections. (D) Heat map showing significantly different gene expression in infection, recovery, and fatal groups compared to the control group. Canonical pathways involved in each gene block are shown on the right side of the heat map. (E) The composition of the B-cell subset in each group is shown as percentages. Each colored bar indicates a different B-cell subset. (F) Volcano plot showing differentially expressed genes in the SFTSV^+^ B cells (red in panel B) versus SFTSV^−^ B cells (gray in panel B) in the fatal group. Selected upregulated genes are shown in red, and selected downregulated genes are shown in blue. The viral N mRNA is not shown in the plot because it is above the upper range of the *P* value. (G) Differential expression pathways of SFTSV-infected B cells versus noninfected B cells in lethal SFTSV infections are graphed with their activation z scores. Activation z score below zero indicates pathway inactivation, while score above zero indicates pathway activation. (H) Violin plots showing top four genes from each canonical pathway enriched in SFTSV^+^ B cells compared to SFTSV^−^ B cells. Statistically significant gene expression is observed only if a violin-shaped fitting area can be calculated.

Aligning our scRNA-seq results with a custom genome, which included both human and SFTSV sequences, allowed us to identify cells that harbor viral mRNA. Our results showed that SFTSV transcripts (RNA-dependent RNA polymerase [RdRp] [L segment], Gn/Gc [M segment], and N/NSs [S segment]) were detected only in the fatal group and not in the infection or recovery group. Approximately 23 cells from the fatal group harbored one or more viral transcripts (Fig. 4A). These viral mRNAs (Fig. S3A) were detected in two out of three fatal cases with higher viral titers in the sera (Fig. 2B). Surprisingly, 22 out of the 23 viral transcript-carrying cells were annotated as B cells, with the remaining cell being a T cell (Fig. 4B and Fig. S3B). By subclustering the B-cell population, we identified naive B cells (MME/CD10^+^ CD52^++^), memory B cells (MS4A1/CD20^++^ CD22^++^), plasmablasts (CD27^++^ TNFRSF17/CD269^+^), and plasma cells (CD27^++^ IGTA4^+^ SDC1/CD138^+^) based on their specific markers as previously described (Fig. S3C) (7, 23–25). Almost all of the infected B cells were plasma cells except for one cell identified as a plasmablast (Fig. 4C). These results demonstrate that the SFTSV replication level is highly correlated with disease severity and that B cells, specifically plasma cells, are a potential viral reservoir in the blood of fatal SFSTV infections.

10.1128/mBio.02583-20.3 FIG S3 Detection of SFTSV transcripts in B cells. (A) PBMC samples from fatal 1 and fatal 3 samples containing SFTSV transcripts. The table shows the barcode of each viral transcript identified within each patient sample, the specific barcode-corresponding SFTSV gene, and the number of each transcript within a target cell. (B) Dot plot of gene expression markers for B cells, T cells, NK cells, monocytes, macrophages, and dendritic cells. CD14 is a common marker shared between monocytes and macrophages. The color of each dot represents the expression level of a specific gene, while the size indicates the percentage of cells expressing each specific gene. Gray indicates very low to undetectable expression. (C) Dot plot showing the gene expression profile for each B-cell subtype: naive, plasma cells, memory cells, and plasmablasts. This categorization is applied to all sample groups to identify B-cell composition. (D) The absolute numbers of naive B cells, plasma B cells, memory B cells, and plasmablasts for each sample group. (E) Heat map showing normalized expression of the top gene sets in B cells that define each patient group. Within each patient group, the B cells are further divided into the four B-cell subtypes. Left margin color bars highlight gene sets specific to each infection group (in the order of control, infection, recovery, and fatal). Download FIG S3, PDF file, 0.8 MB. Copyright © 2021 Park et al. 2021 Park et al. https://creativecommons.org/licenses/by/4.0/ This content is distributed under the terms of the Creative Commons Attribution 4.0 International license.

To identify the transcriptional signatures associated with SFTSV infection in the B-cell population, we averaged the total gene expression in B cells of each infection group and compared it to that of the control group (Fig. 4D). In the fatal group, total B cells showed a broad induction of the IFN and sirtuin signaling pathways, with a reduction in B-cell development, antigen presentation, immune cell communication, and IL-4 signal pathways. In addition, we observed significant compositional changes in cell populations (Fig. 4E) and gene expression patterns in B-cell subtypes in each infection group (Fig. S3D and E). The fatal group showed a dramatic induction of the plasmablast population, a reduction of the plasma cell population, and nearly complete depletion of the naive B-cell population.

In order to determine the effect of SFTSV infection on B-cell-mediated immune responses, we identified genes that were enriched or reduced in infected B cells compared to uninfected B cells within the fatal group. A volcano plot shows that 420 genes were upregulated (e.g., FEZ1, IL-21R, EBI3 [IL-27β], TRAF4, and IGHG1) and 111 genes were downregulated (e.g., ISG15, IFI27, IFI6, and OAS1) in infected B cells compared to uninfected B cells from the fatal group (Fig. 4F). Pathway enrichment analysis revealed strong suppression of sirtuin and IFN signaling pathways in infected B cells compared to the uninfected B cells in the fatal group (Fig. 4G and H). This indicates that while sirtuin and IFN signaling pathways are highly induced in the total B-cell population of the fatal group (Fig. 4D), in the SFTSV-infected B-cell population these pathways are specifically reduced relative to the uninfected cells (Fig. 4G). On the other hand, the NRF2-mediated oxidative stress response, B-cell receptor, and phosphatidylinositol 3-kinase (PI3K)/AKT signaling pathways were activated in infected B cells, but not in uninfected B cells, of the fatal group (Fig. 4G and H). When we compared the DEGs of infected plasma cells to those of uninfected plasma cells from the fatal group, a similar trend was observed, with downregulation of IFN and sirtuin signaling pathways and upregulation of the NRF2-mediated oxidative stress response (Fig. S4A and B). These results indicate that B cells, specifically plasma cells, are potential viral reservoirs in the blood during fatal SFTSV infections. Also, while cases that result in fatalities in general display high upregulation of the IFN signaling pathway and perturbation of B-cell development, individual SFTSV-infected B cells show the opposite, downregulation of IFN signaling and upregulation of B-cell receptor signaling.

10.1128/mBio.02583-20.4 FIG S4 Differential gene expression between SFTSV^+^ and SFTSV^−^ plasma B cells in patients who died of SFTSV infection. (A) Volcano plot showing differentially expressed genes in the fatal SFTSV^+^ plasma B cells compared to the fatal SFTSV^−^ plasma B cells. Selected upregulated genes are shown in red, and selected downregulated genes are shown in blue. The SFTSV N transcript is not shown because it was above the upper range of the y axis *P* value. (B) Differential gene expression pathways of fatal SFTSV^+^ plasma B cells versus fatal SFTSV^−^ plasma B cells are graphed with their activation z score. An activation z score below zero indicates inactivation of the pathway, while a score above zero indicates activation. Download FIG S4, TIF file, 0.2 MB. Copyright © 2021 Park et al. 2021 Park et al. https://creativecommons.org/licenses/by/4.0/ This content is distributed under the terms of the Creative Commons Attribution 4.0 International license.

### B cells are the primary target of *in vitro* SFTSV blood infection.

**FIG 5:**
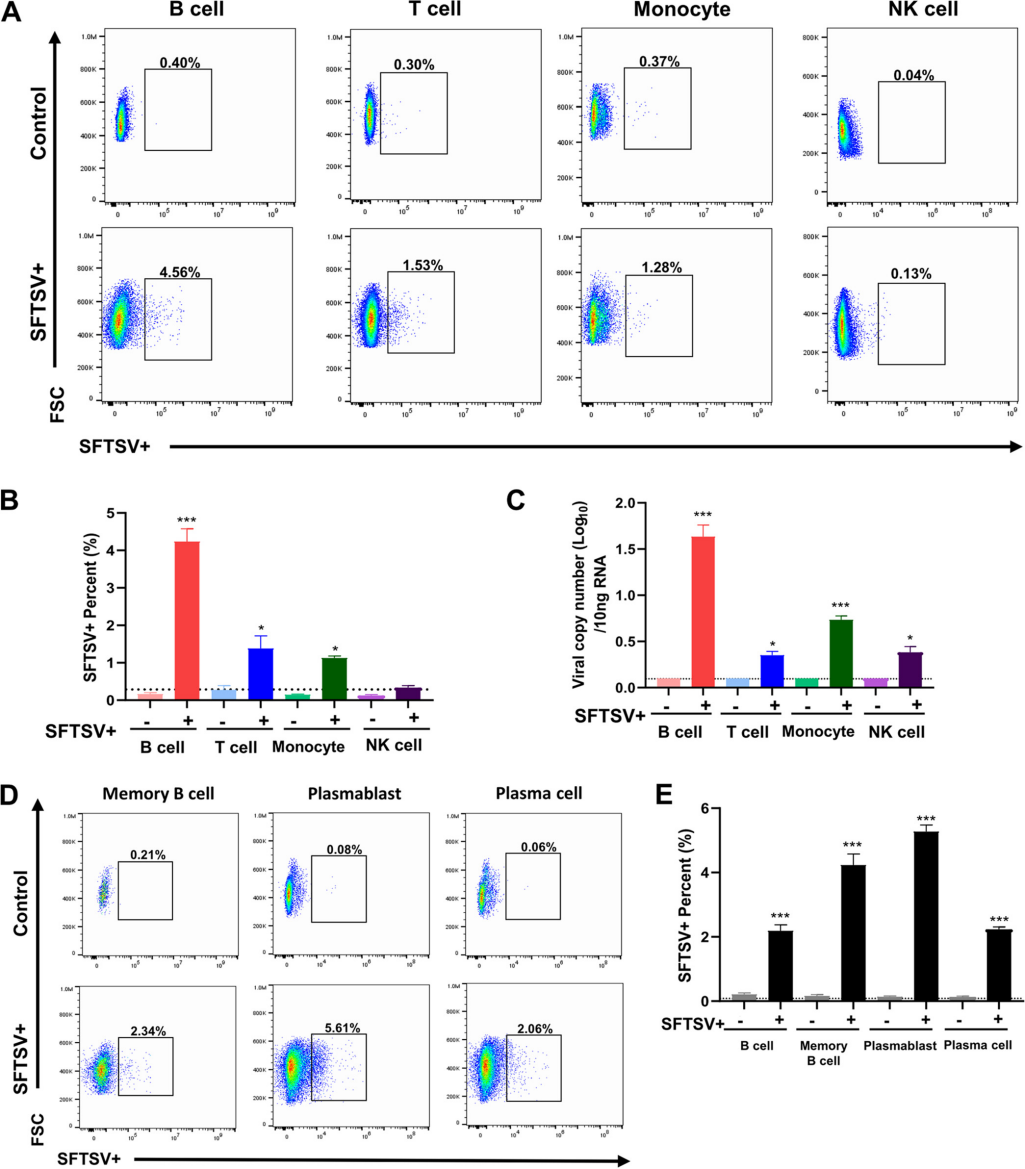
High susceptibility of B cells to *in vitro* SFTSV infection. Whole blood from healthy donors (*n *= 6) was infected with SFTSV strain CB1/2014 or serum-free medium (control) at MOI 1 conditions (5% CO_2_, 37°C). After 48 h of infection, red blood cells were removed by lysing in red blood cell lysis buffer (Thermo Fisher Sciences, USA) and then SFTSV infection of each cell subset was assessed by intracellular staining with anti-SFTSV N antibody (in-house monoclonal antibody). (A) CD19^+^ B cells, CD3^+^ T cells, CD14^+^ monocytes, and CD56^+^ NK cells were identified in SFTSV-infected PBMCs by FACS with subset-specific antibodies. The percent change of each SFTSV-infected PBMC subset (lower row) compared with that of noninfected control PBMC subset (upper row) is presented. (B) Average percentage of PBMC subsets infected with SFTSV in six donors. The noninfected control for each PBMC subset was less than 1% in all donors. (C) Virus copy numbers in each sorted PBMC subset (CD45^+^ CD19^+^ B cells, CD45^+^ CD3^+^ T cells, CD45^+^ CD14^+^ monocytes, and CD45^+^ CD56^+^ NK cells) were measured using qRT-PCR with primers specific for the M gene. (D) Detailed subsets of SFTSV-infected B cells were identified using FACS (CD19^+^ CD27^+^ [memory B cell], CD19^+^ CD20^−^ CD38 [plasmablast], and CD20^−^ CD38^+^ CD138 [plasma cell]) and an SFTSV N antibody and are presented as the percent change. (E) Average percentage of each B-cell subset infected with SFTSV in all donors (*n *= 6). Noninfected control for each B-cell subset was less than 1% in all donors. Asterisks indicate statistical significance between the control and infection groups as indicated by the line, determined by one-way ANOVA and subsequent Tukey’s test (*, *P < *0.05; **, *P < *0.01; ***, *P < *0.0001).

To further confirm that B cells are the potential target of SFTSV blood infection, we performed infections of whole blood obtained from healthy donors (*n *= 6) using SFTSV CB1/2014 at a multiplicity of infection (MOI) of 1. At 48 h postinfection (hpi), PBMCs were isolated from whole blood specimens and the susceptibility to SFTSV infection was determined using fluorescence-activated cell sorting (FACS) and viral load qRT-PCR. To identify the potential target cells of SFTSV infection, we sorted and gated the isolated PBMCs by flow cytometry into four immune subsets (B cells, T cells, monocytes, and NK cells) (Fig. S5), followed by detection of SFTSV by M-specific reverse transcription-quantitative PCR (qRT-PCR) and anti-nucleocapsid (anti-N) protein intracellular antibody staining. Upon infection, we did not see significant changes in the cellular composition compared to that of the uninfected control. While each cell type showed variable susceptibility to SFTSV, both viral load and FACS analyses identified B cells as the primary target for *in vitro* SFTSV infection and replication (Fig. 5A to C). To determine the subset of B cells most susceptible to SFTSV infection, we used surface markers to distinguish memory B cells (CD19^+^ CD20^+^ CD27^+^), plasmablasts (CD19^+^ CD20^−^ CD38^+^), and plasma cells (CD19^+^ CD20^−^ CD38^+^ CD138^+^) as previously described (26). The results showed that while all three B-cell subsets are highly susceptible to SFTSV infection, plasmablasts showed higher infection rates than other subsets (Fig. 5D and E). These results demonstrate that B cells are the primary targets for *in vitro* SFTSV blood infection.

10.1128/mBio.02583-20.5 FIG S5 Flow cytometry gating strategy to define PBMC subsets. Shown is a flow cytometry gating strategy to identify four different subsets in SFTSV-infected whole blood. Forward scatter (FSC) and side scatter (SSC) gating strategy was used to obtain T cells, B cells, and NK cells based on size and lineage-specific markers of each lymphocyte. Lineage-specific antibodies against T cells (CD3^+^), B cells (CD19^+^), and NK cells (CD56^+^) were used to identify specific PBMC subsets. Additionally, the monocyte population was gated with CD14^+^ specific antibody. B-cell subsets, including memory B cells (CD19^+^ CD27^+^), plasmablasts (CD19^+^ CD20^−^ CD38^+^), and plasma B cells (CD19^+^ CD20^−^ CD38^+^ CD138^+^), in PBMCs were assessed using flow cytometry with specific antibodies. Download FIG S5, TIF file, 0.4 MB. Copyright © 2021 Park et al. 2021 Park et al. https://creativecommons.org/licenses/by/4.0/ This content is distributed under the terms of the Creative Commons Attribution 4.0 International license.

## DISCUSSION

In this study, we aimed to understand the impact of SFTSV infection on circulating immune cells of SFTS patients at different disease states by examining their immune signaling profiles and single-cell gene expression. This study not only identified novel molecular signatures of the serum inflammatory profile and B-cell lineage populations but also distinguished B cells, notably plasma B cells, as one of the potential viral targets in blood of fatal SFTSV infection. This immune landscape study of SFTS patient blood indicates that SFSTV targets the B-cell lineage and alters the function of these cells, which may contribute to lethal viral infection.

Within the 76 inflammatory molecules identified in SFTS patient sera, several signaling pathways and specific inflammatory biomarkers were found to be associated with severe SFTS cases. Particularly, serum immunoprofiling showed distinct clustering of different inflammatory factors in low-risk and high-risk groups, wherein all fatal cases were included in the high-risk group. The marked induction of proinflammatory cytokines and chemokines in fatal cases was likely the result of immune cell infiltration. We also found that increased expression of caspase-8, CCL7, SIRT2, CXCL9, STAMBP, CCL20, and 4EBP1 were highly associated with severe cases of SFTS disease, while TNF-α, CCL20, and CX3CL1 cytokine levels were specifically associated with fatality. A previous study (12) also showed that an increase of the acute systemic inflammatory cytokine TNF-α in serum is associated with SFTS disease severity. Moreover, CCL20 is strongly chemotactic for lymphocytes and neutrophils and its expression can be induced by inflammatory cytokines such as TNF-α. This study suggests that these soluble inflammatory factors may cooperate to induce strong, acute inflammation. Further, these inflammatory cytokines may be useful biomarkers to identify patients at risk of developing severe symptoms and potential fatality after disease onset. However, since we have not done a follow-up study, we would not know whether these potential biomarkers can be applied to other stages of the disease. In addition, as our study was a pilot observation of a small group of patients, additional studies with larger groups of patients are needed.

Previous studies (7, 27) have evaluated PBMC subsets of SFTSV-infected patients using flow cytometric analysis or qRT-PCR of sorted populations. B cells have been shown to play a crucial role in determining disease severity. For example, overactivation and exhaustion of naive B cells and overproliferation of plasmablasts were previously characterized in severe SFTS patients. In addition, it has recently been suggested that B cells are targets of SFTSV lethal infection, as postmortem analysis of lymph nodes identified the majority of SFTS-infected cells as B cells, specifically plasmablasts (28). Thus, our study is consistent with the previous findings showing the crucial role of B cells in fatal SFTSV infections. While a previous immunohistochemical analysis of secondary lymph nodes from SFTSV-lethal cases (28) showed that plasmablasts primarily carry SFTSV infection, our scRNA-seq subtyping analysis of PBMCs of SFTSV-infected patients indicated that plasma cells mainly harbored viral RNAs. The differences in specific B-cell subsets may be due to the different sources of SFTSV-infected tissue samples, for instance, lymph nodes versus PBMCs. After leaving secondary lymph nodes, plasmablasts, which are dividing antibody-secreting cells, migrate to the bone marrow, where they are exposed to survival factors necessary to finally differentiate into long-lived plasma cells (29). These plasma cells then circulate in the blood, where they can produce antibodies. We speculate that plasmablasts may be initially infected with SFTSV in lymph nodes and then differentiated into plasma cells that subsequently circulate in the blood. On the other hand, it is also possible that SFTSV independently infects plasmablasts in lymph nodes and plasma cells in the blood. Finally, it should be noted that while it is known that most bunyavirus mRNAs are not polyadenylated during replication, we identified internal alternative polyadenylation sites within L, M, and S segments of the SFTSV genome (Fig. S6) that were potentially used for scRNA-seq analysis. These internal alternative polyadenylation sites are also in agreement with a previous work showing that hantaviruses contain similar polyadenylation stretches at their transcription termination sites (30). Nevertheless, additional studies are needed to fully characterize *in vitro* and *in vivo* B-cell infection of SFTSV.

10.1128/mBio.02583-20.6 FIG S6 Potential alternative internal polyadenylation sites in SFTSV genome. The L, M, and S segments of SFTSV genome are shown in Integrated Genomics Viewer (IGV). From top to bottom, SFTSV transcripts, adenine-rich regions, and polyadenylation hexamers are shown on the IGV tracks. Putative internal polyadenylation sites are highlighted in the red boxes. Download FIG S6, TIF file, 0.2 MB. Copyright © 2021 Park et al. 2021 Park et al. https://creativecommons.org/licenses/by/4.0/ This content is distributed under the terms of the Creative Commons Attribution 4.0 International license.

SFTSV-infected B cells demonstrated a significant reduction of IFN and sirtuin signaling pathways relative to those of uninfected B cells. On the other hand, the entire B-cell population of the fatal group showed elevated IFN and sirtuin signaling compared to the control group. We suggest that the reduction of IFN signaling in SFTSV-infected B cells is due to the IFN-antagonistic function of the viral NSs protein. We and others have shown that viral NSs protein effectively inhibits type I and II IFN signaling pathways to suppress ISG expression and IFN-γ production (31–34). Furthermore, inhibition of sirtuin has been shown to increase influenza A virus titers (35). This suggests that while uninfected immune cells attempt to increase IFN and sirtuin pathways to prevent virus infection and replication, SFTSV-infected B cells may sufficiently block the paracrine effect on neighboring uninfected cells, allowing high viral replication. Thus, the detailed molecular mechanisms underlying the tug of war between infected and uninfected cells need to be studied further.

While the scRNA-seq analysis of SFTS patient blood provides molecular details of SFTSV infection in patients, there are limitations to our study underscoring the need for further analysis. First, the scRNA-seq analysis in this study included a relatively small sample number (10 patient samples), while the immune profiling analysis had a considerable sample size (51 serum specimens). To further support our results, a larger number of patient samples should be required. However, despite the small sample size, we were able to identify the novel molecular signature of the B-cell lineage population in fatal SFTSV infections. Second, scRNA-seq failed to detect viral transcripts in the infection group, possibly due to the low capturing efficiency of the platform from 10x Genomics and/or the presence of viral transcripts below the detection level. Third, the present study data did not have a high enough resolution to characterize the less prominent immune cell populations, such as classical/nonclassical monocytes and mast cells, which may also be involved in SFTSV infection. Thus, additional studies are needed for the molecular details of SFTSV infection in patients, while previous and our studies suggest that B cells appear to harbor the large amount of SFTSV during fatal infection. For instance, a combination of immunohistochemistry, flow cytometry, scRNA-seq, and cellular indexing of transcriptomes and epitopes by sequencing (CITE-seq) (36) may not only determine the detailed cell population carrying SFTSV but also identify potential changes of infected cells upon SFTSV infection.

In summary, by combining the proximity extension assay and single-cell transcriptome analysis, we provide the first comprehensive analysis of the dynamic immune landscape in SFTS patients. We show that during fatal infections, SFTSV causes an excessive inflammatory response through marked induction of proinflammatory cytokines and chemokines and the aberrant inactivation of the adaptive immune response. Furthermore, the majority of SFTSV in fatal infections was found in plasma B cells. Thus, SFTSV infection may inhibit high-affinity antibody maturation and secretion of plasma B cells, suppressing neutralizing antibody production and thereby allowing significant virus replication and subsequent fatality. Further study is needed to prove or disprove this hypothesis.

## MATERIALS AND METHODS

### Human subjects and blood collection.

Blood samples were collected from patients suspected to have SFTS and patients who had recovered from SFTS between 2014 and 2019. All work with human subjects was approved by the Chungbuk National University Hospital (IRB approval number 2017-05-002-001) and Asan Hospital (IRB approval number 2016-0046). PBMCs were extracted using Ficoll-Paque PLUS (GE Healthcare, Uppsala, Sweden) and stored at −80°C until use. The samples were collected right after hospitalization, and samples from the recovery group were collected at least 2 weeks postdischarge. Serum aliquots from these patients were stored at −80°C until the analysis.

To determine if B cells are the primary target for *in vitro* SFTSV blood infection, peripheral blood specimens were obtained from healthy donors (*n = 6*) and stored temporarily at 37°C in a lithium heparin-containing tube (BD) prior to infection.

### Virus propagation and titration.

To infect cells, viruses were propagated in Vero E6 cells in Dulbecco modified Eagle medium (DMEM; Gibco) supplemented with 1% penicillin/streptomycin (Gibco) and 2% fetal bovine serum (FBS; Gibco) in a 37°C incubator supplemented with 5% CO_2_ for 6 days. The propagated virus was stored at −80°C as the working virus stock for animal studies. Viral infectivity titers were determined using an immunostaining assay in which the 50% tissue culture infective dose (TCID_50_) was determined with an in-house-generated monoclonal N antibody against SFTSV.

### Plasma protein quantification using proximity extension assay (PEA).

Serum specimens from control healthy participants (*n *= 6), SFTSV-infected patients (*n *= 40), patients that succumbed (*n *= 11), and fully recovered patients (*n *= 5) were collected from SFTSV-infected patients in hospitals throughout South Korea between 2014 and 2019. The serum was centrifuged at 12,000 rpm for 5 min at 4°C and stored at −80°C until analysis. Plasma protein quantification data were generated using Olink’s inflammation panel (Olink AB, Uppsala, Sweden), which allowed quantification of unique proteins from less than 5 μl of plasma. An inflammatory panel of 92 proteins was used to detect different markers in the sera collected from the cohort with a method that has been described previously (37). Briefly, Olink probes with DNA oligonucleotide-labeled antibodies bind to proteins in the sample, and when two antibodies are brought into close proximity due to binding to the same target, their DNA sequences overlap; these are then quantified using real-time PCR. Of the 60 tested sera, 51 samples (5 control, 8 fatal, 35 infection, and 3 recovery) passed quality control and were subjected to further analyses, and out of 92 proteins, 77 passed the 80% detection limit.

### PEA computational analysis.

Cytokines with values below the lower limit of detection (LLOD) were replaced by the half value of the limit of detection (LOD). Similarly, missing values were replaced with the median value of cytokine level between samples. To classify the patient groups and visualize the association based on the level of cytokines in blood sera, the *t*-distributed stochastic neighbor embedding (*t*-SNE) algorithm was applied for unsupervised clustering using R package *Rtsne* (version 0.15). SFTSV patients were divided into three groups: healthy control (HC), low-risk infection (LRI), and high-risk infection (HRI). Then, the log_2_-transformed cytokine expression data with fold change between different subgroups and healthy control was visualized in a heat map plot using R package *pheatmap* (version 1.0.12).

Pairwise comparisons of levels of each cytokine were performed by Welch *t* test from the R *base* package. For functional interpretation of the induced cytokines in SFTSV-positive patients compared to healthy controls, gene ontology (GO) analysis of the cytokines significantly induced (*P* < 0.05) in SFTSV patients was performed using EnrichR (38, 39). R package ggplot2 (version 3.2.1) was used to plot the comparison of cytokines in different groups (e.g., healthy control/SFTSV^+^ patients, low-risk infection/high risk infection, and fatal cases/other subgroups). Statistical analyses and data processing were performed in R version 3.5.1 (R Foundation for Statistical Computing, Vienna, Austria) and Python version 3.6.7. (Python Software Foundation [https://www.python.org/]).

### Expression of cytokines and chemokines in serum.

A standard multiplex biometric immunoassay was utilized to assay cytokines (Bio-Plex Pro human cytokine assay; Bio-Rad, Hercules, CA). Briefly, diluted serum samples were incubated with antibody-coupled beads corresponding to the various cytokines. After performing washes to remove unbound protein, the beads were incubated with a biotinylated secondary antibody. The reaction mixture was then detected by the addition of streptavidin-phycoerythrin (streptavidin-PE). Assays were measured by detection of the fluorescent reporter signal and identification of beads on a Bio-Plex 200 system, and data were analyzed using the associated Bio-Plex Manager software version 4.1.1 (Bio-Rad).

### Virus detection in patient sera.

Total RNA was extracted from patient sera using TRIzol reagent (Thermo Fisher Scientific) with the standard manufacturer’s protocol, and cDNAs were generated by reverse transcription using QuantiTect (Qiagen). Viral copy numbers were determined by real-time RT-PCR with an M segment-based SFTSV-specific primer set as previously described (43). The forward primer was SFTS-M-F (AATTCACATTTGAGGGTAGTT), and the reverse primer was SFTS-M-R (TATCCAAGGAGGATGACAATAAT). Real-time PCRs were performed using a SYBR green supermix (Bio-Rad) and a CFX96 Touch real-time PCR detection system (Bio-Rad). Copy numbers were calculated as a ratio with respect to the standard control (40).

### scRNA-seq profiling.

PBMCs from healthy controls (control group; 2 males and 1 female, more than 60 years of age; *n *= 3), SFTS patients during infection and post-viral clearance (infection group and recovery groups, respectively; 65-year-old female and 76-year-old male; *n *= 2), and fatal cases (fatal group; 2 males and 1 female, 60 to 73 years of age; *n *= 3) were collected from SFTSV-infected patients in hospitals throughout the Republic of Korea. PBMCs from each individual were processed separately through the Chromium single-cell platform with the 10x Genomics chromium single cell 3′ library and gel bead kit v2 and the Chromium single cell A chip kit (PN-120267) using the standard manufacturer’s protocol. The constructed libraries were sequenced using the HiSeq 4000 (Illumina, San Diego, CA) system in a 2 × 150 bp paired-end with an average of 50,000 reads per cell. The results were demultiplexed by Theragen Etex Inc. (Republic of Korea), which provided the *fastq* files and sequencing quality statistics. Each individual’s data in each group were bioinformatically merged. Each group contained 4,500 to 11,000 cells, and in total, 25,799 cells were analyzed.

### scRNA-seq computational analysis.

A custom reference genome that combines the hg38 human reference genome and the SFTSV KASJH reference genome (accession numbers KP663731 to KP663733) (41) was generated and used for alignment. Initial alignment and analysis were done using Cell Ranger v2.1.0 from 10x Genomics. The resulting filtered gene matrices were then passed to the Seurat R package v3.1 for further preprocessing (42). Dead cells were removed by excluding cells with a mitochondrial gene content greater than 10% of the total unique molecular identifier (UMI) count. Cells were then normalized by dividing the UMIs of each gene by the total UMIs within each cell, multiplying the resulting scale factor by 10,000, and then log-transforming the final result. Cells were initially preprocessed by patient sample and then combined for further analysis.

After the preprocessing, the combined cells were scaled so that the mean expression for every gene across cells was 0, while the variance of the gene expression was 1. Genes exhibiting high cell-to-cell variation within our data set were then identified and used for linear dimensional reduction with principal-component analysis (PCA). Based on the PCA scores, the principal components 1 to 7 for nonlinear dimensional reduction and unsupervised clustering were used. Data visualization was done with the *t*-distributed stochastic neighbor embedding (*t*-SNE) method. All data for heat maps, *t*-SNE plots, violin plots, and dot plots were produced using Seurat functions. For clustering, the standard FindClusters function in Seurat with the default Louvain algorithm was used. For pathway analysis, differential gene expression was first generated from Seurat and imported into Microsoft Excel. The differential gene expression only contained genes with a fold change of greater than 2 or less than −2 and a *P* value of <0.05.

### Flow cytometric analysis.

To identify the primary target cells of SFTSV infection (MOI = 1), PBMCs from infected whole blood were isolated 48 h postinfection (hpi) and specific immune subsets were gated (B cells, T cells, monocytes, and NK cells). All antibodies and reagents used in flow cytometric analyses were purchased from Thermo Fisher Scientific (USA) and BD Science (USA), and the antibodies were grouped into six panels for the phenotypic analysis of B cells (a to c), T cells (d), monocytes (e), and NK cells (f). Red blood cells were removed by lysing in red blood cell lysis buffer. Antibody combinations in each panel was as follows: panel a, CD19-PE-Cy7, CD20-allophycocyanin (APC), and CD27-PE; panel b, CD19-PE-Cy7, CD20-APC, and CD38-PE; panel c, CD19-PE-Cy7, CD20-APC, CD38-PE, and CD138-SB600; panel d, CD3-PE, CD4-BV711, CD8-BV510, and CD45-APC; panel e, CD14-PE-Cy7 and CD16-APC; and panel f, CD3-Alexa Fluor 700 and CD56-BV421. Intracellular staining of IgG was performed at room temperature for 30 min following cell fixation and permeabilization with FIX/PERM buffer. Immunophenotyping was carried out on an Attune NxT flow cytometer (Thermo Fisher Sciences, USA) and analyzed on FlowJo v10.6.2.

### Statistical analysis.

The statistical significance of hematological analysis compared with naive samples was assessed by two-way analysis of variance (ANOVA) with Tukey’s multiple-comparison test. For comparison of the significance of viral copy numbers or cytokines among samples, we used the one-way ANOVA with Tukey’s multiple-comparison test.

Data plotting, interpolation, and statistical analyses were performed using GraphPad Prism 8.3.1 (GraphPad Software, La Jolla, CA). Statistical details of experiments are described in the figure legends. A *P* value less than 0.05 is considered statistically significant.

### Data availability.

The scRNA-seq data sets generated during this study are available at NCBI GEO database under accession number GSE149313.
