# Supplementary figures and images for: Molecular Signatures of Inflammatory Profile and B-Cell Function in Patients with Severe Fever with Thrombocytopenia Syndrome
Source: mBio. 2021 Feb 16;12(1):e02583-20. doi: 10.1128/mBio.02583-20 (PMC8545090; doi:10.1128/mBio.02583-20)

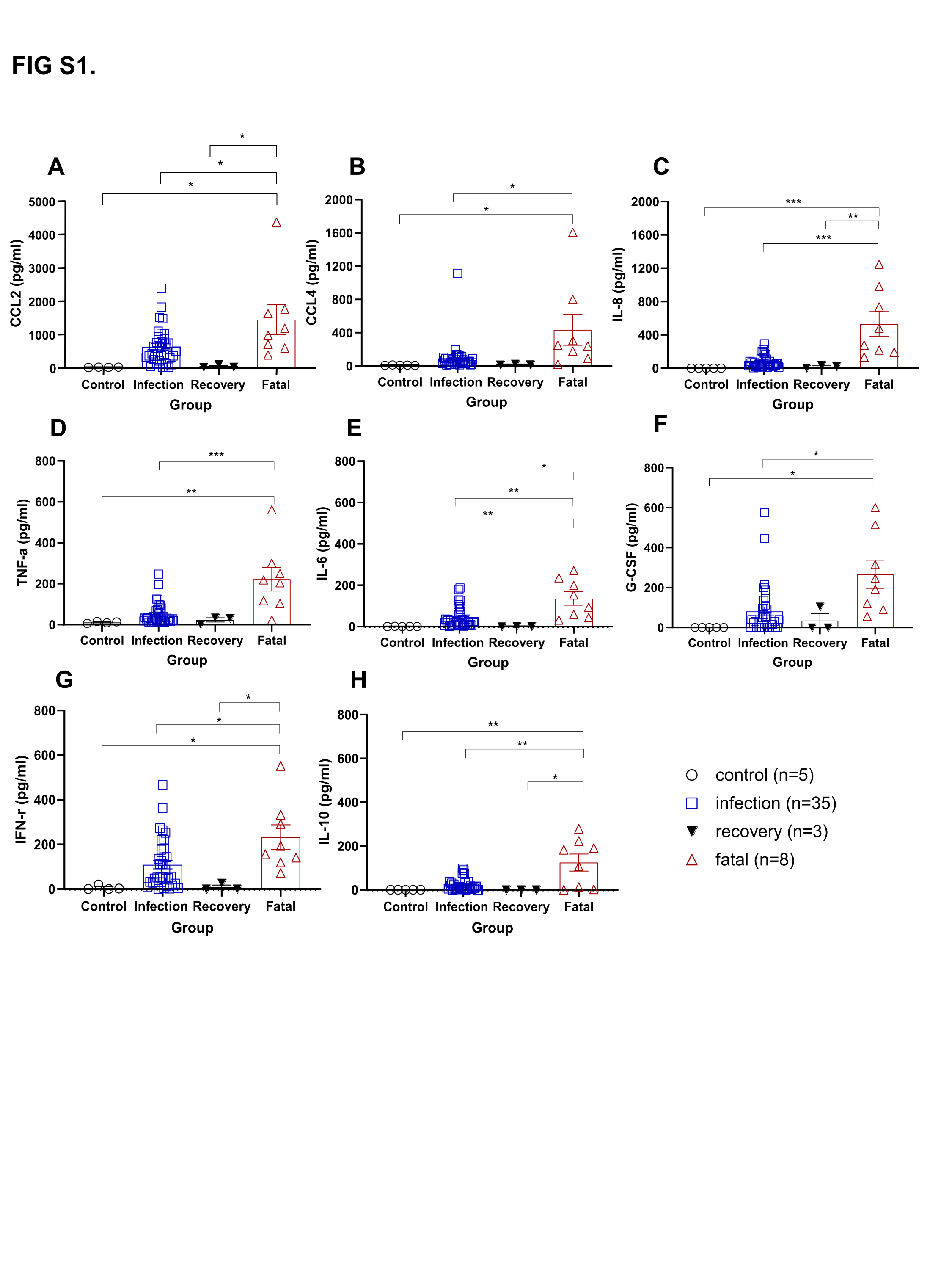

Supplement: FIG S1 [file mbio.02583-20-sf001.tif]

FIG S2.

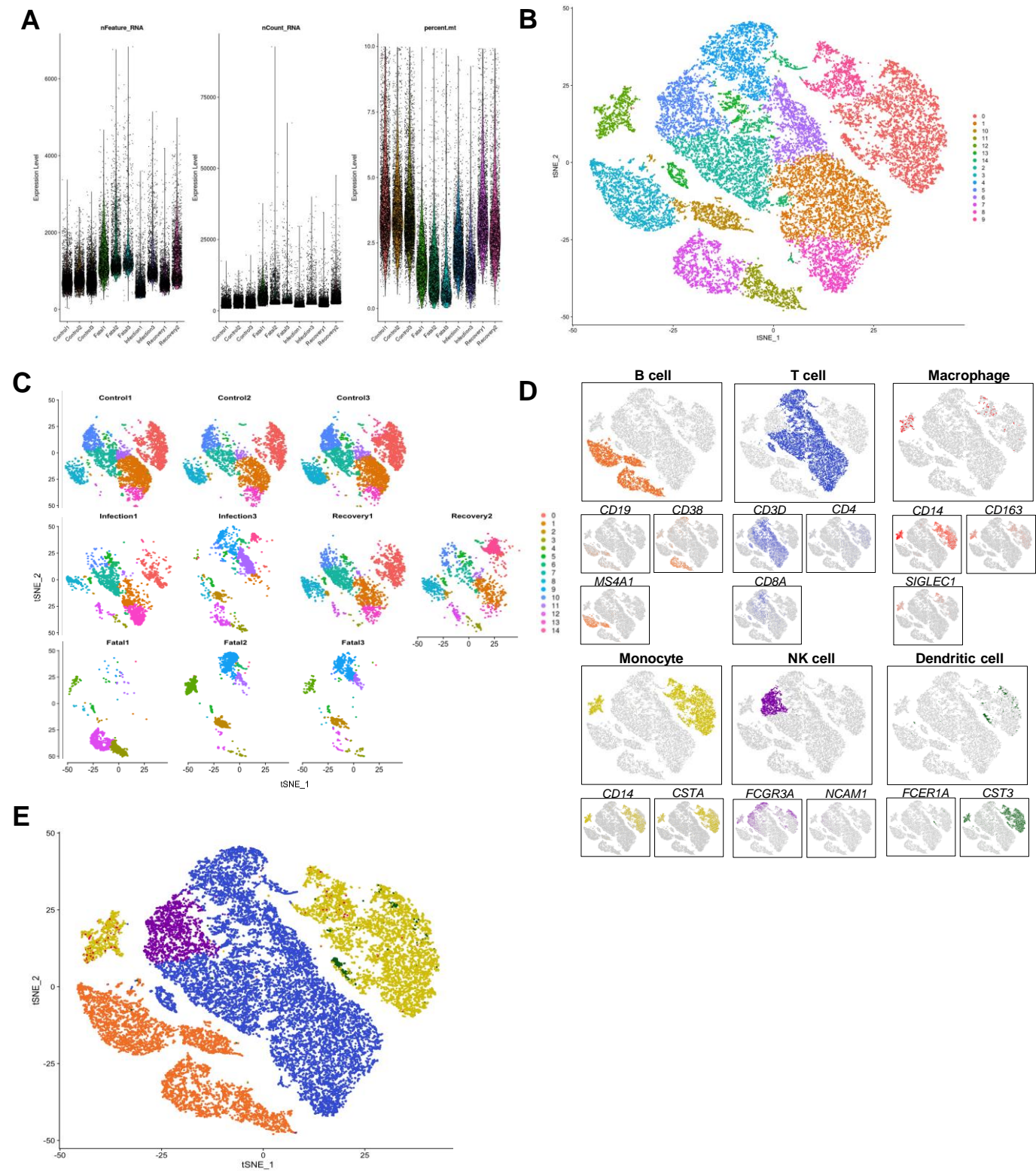

Supplement: FIG S2 [file mbio.02583-20-sf002.pdf]

FIG S3.

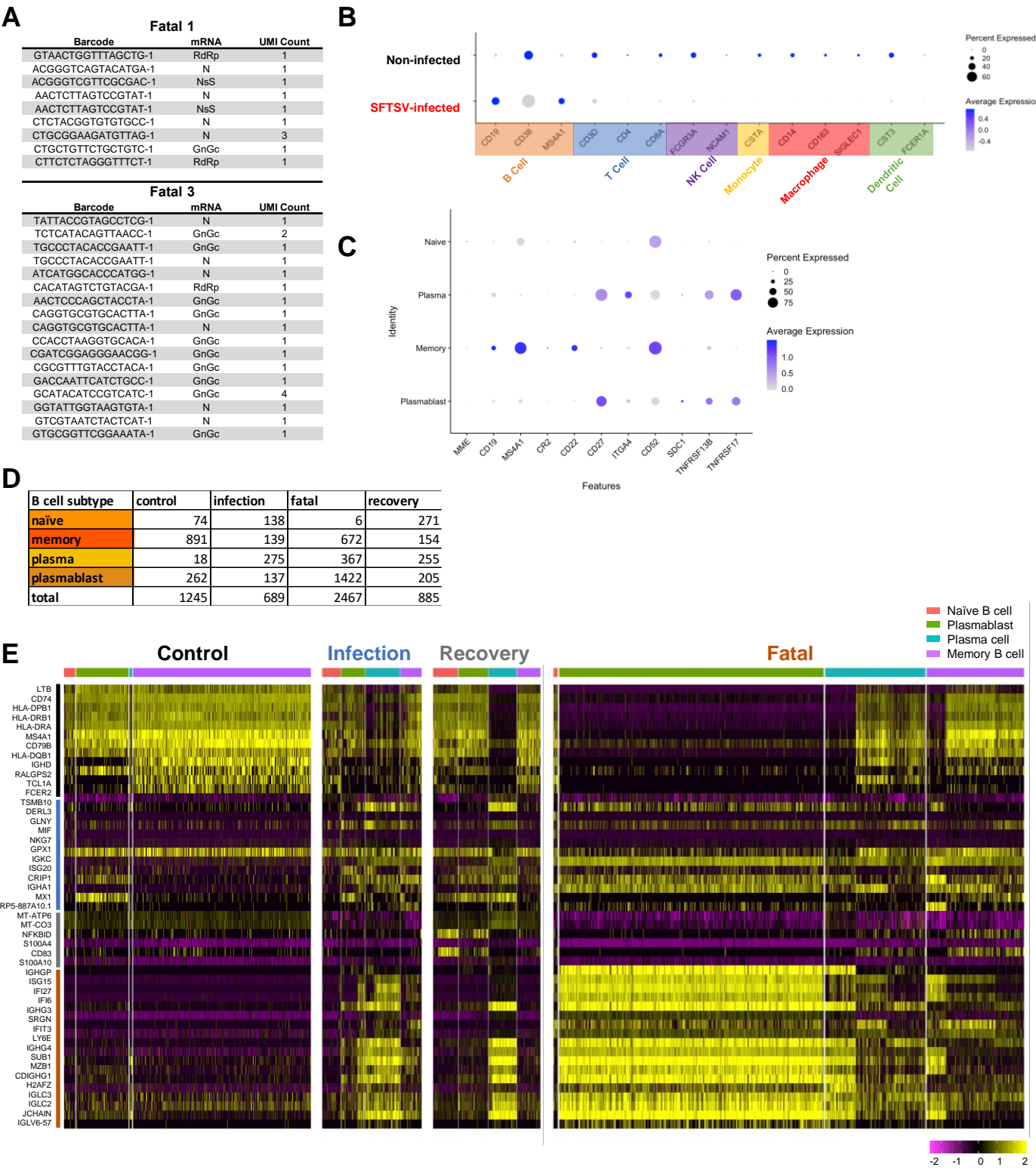

Supplement: FIG S3 [file mbio.02583-20-sf003.pdf]

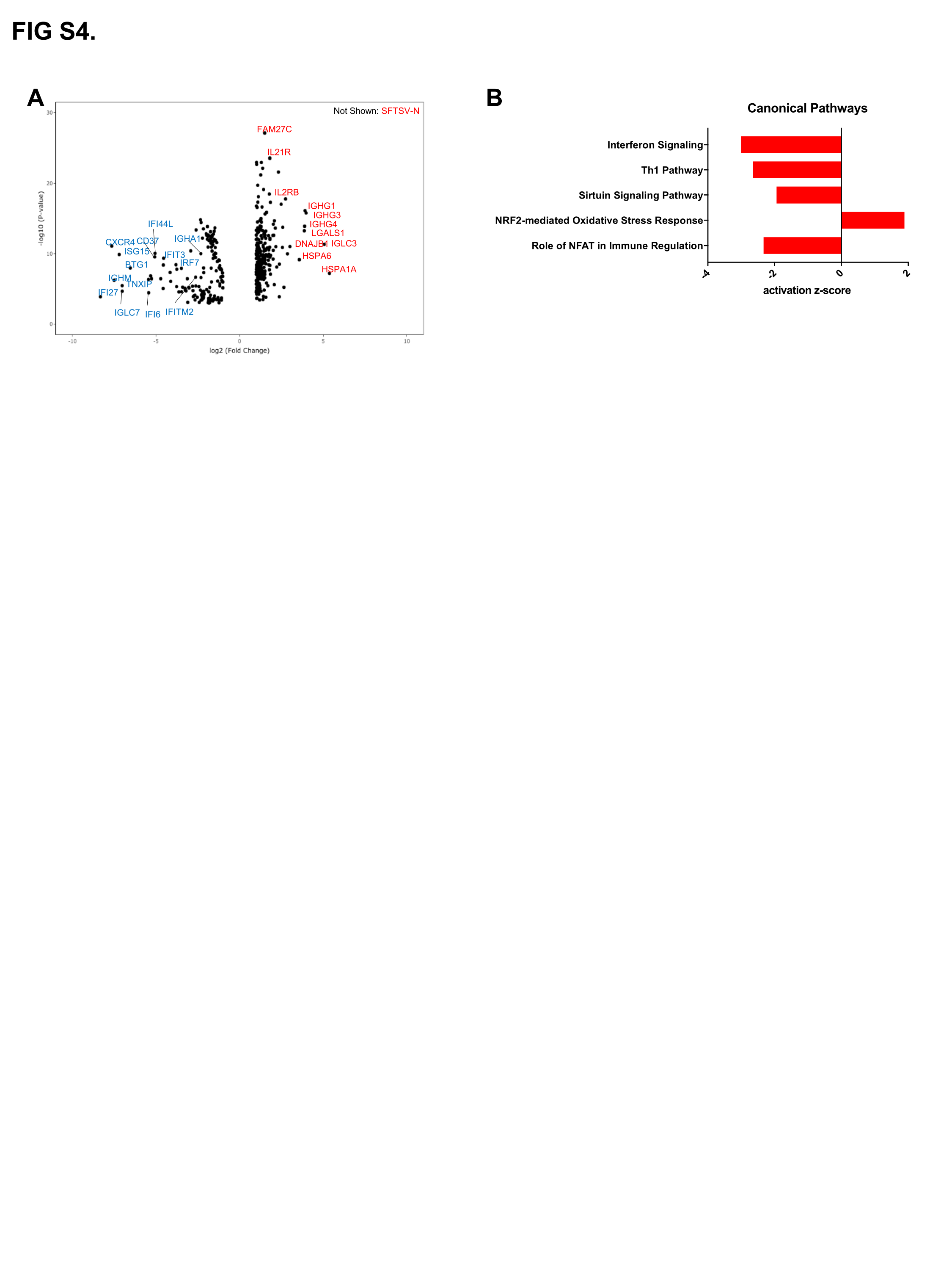

Supplement: FIG S4 [file mbio.02583-20-sf004.tif]

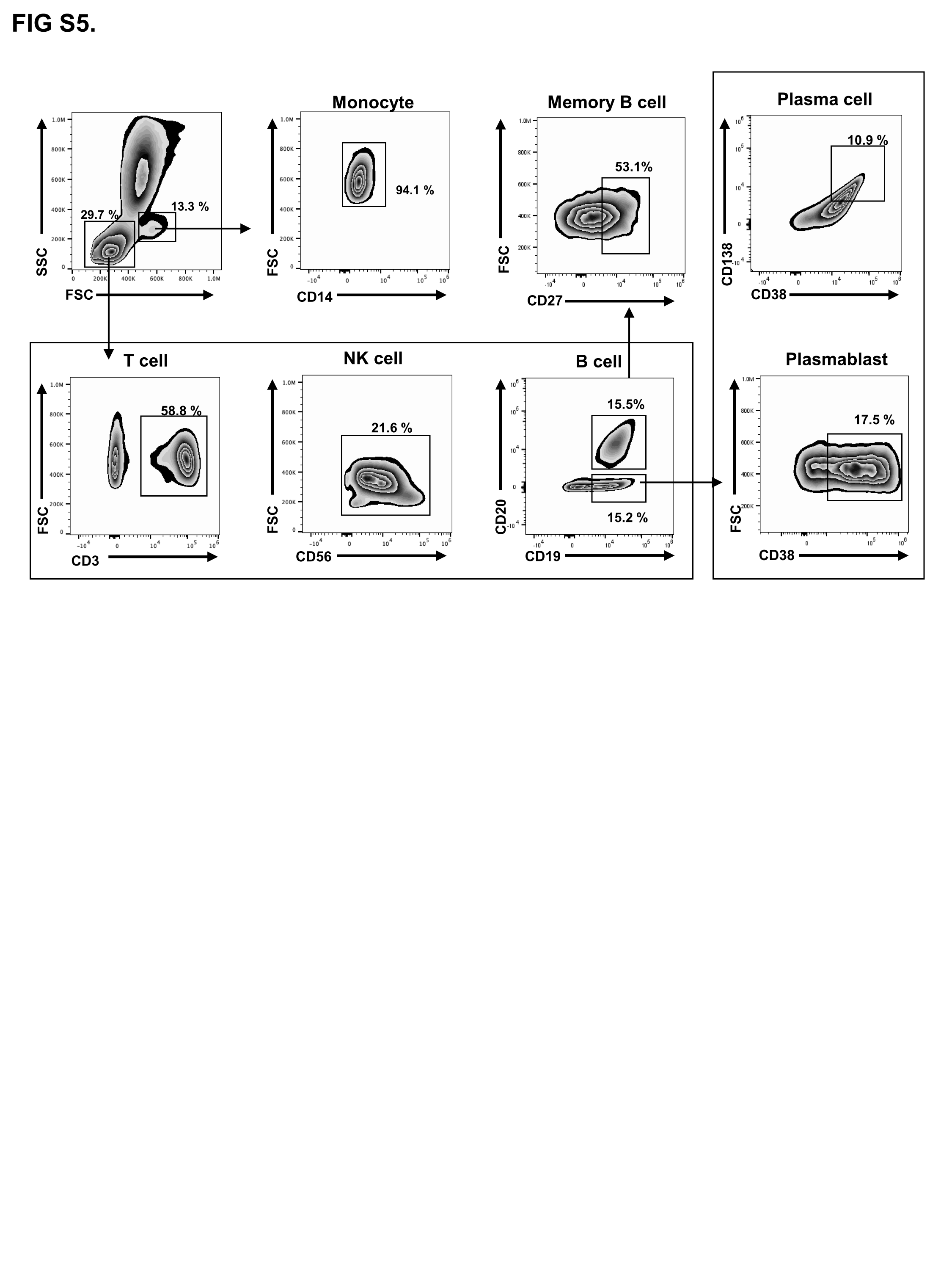

Supplement: FIG S5 [file mbio.02583-20-sf005.tif]

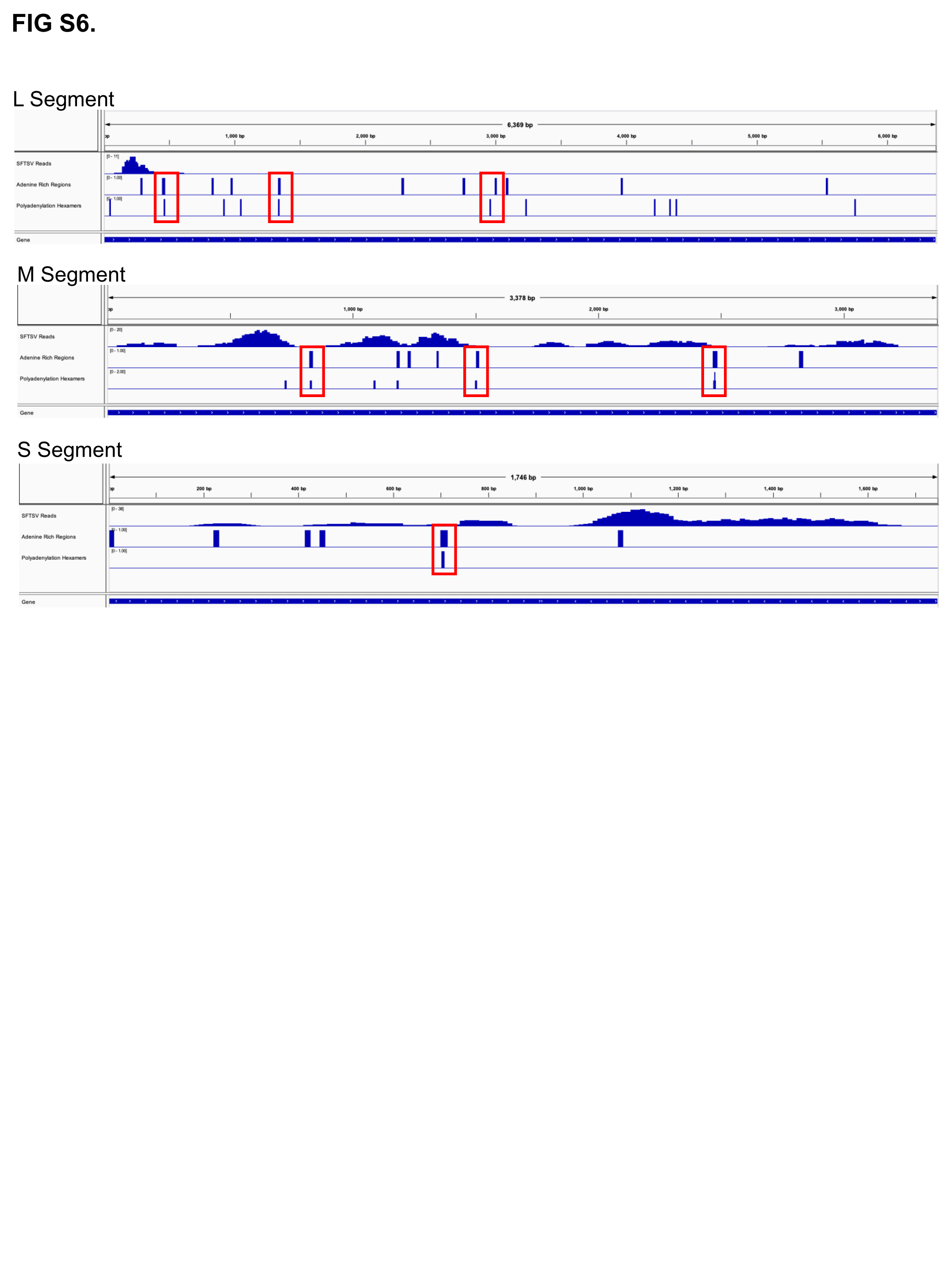

Supplement: FIG S6 [file mbio.02583-20-sf006.tif]
